# pH/Redox/Lysozyme-Sensitive Hybrid Nanocarriers With Transformable Size for Multistage Drug Delivery

**DOI:** 10.3389/fbioe.2022.882308

**Published:** 2022-04-11

**Authors:** Zhe Liu, Dong Zhou, Lan Liao

**Affiliations:** ^1^ The Affiliated Stomatological Hospital, Nanchang University, Nanchang, China; ^2^ The Key Laboratory of Oral Biomedicine, Nanchang, China; ^3^ Jiangxi Province Clinical Research Center for Oral Diseases, Nanchang, China; ^4^ College of Chemistry, Nanchang University, Nanchang, China

**Keywords:** nanocarrier, transformable size, multistage drug delivery, triple sensitivity, antitumor

## Abstract

The majority of current nanocarriers in cancer treatment fail to deliver encapsulated cargos to their final targets at therapeutic levels, which decreases the ultimate efficacy. In this work, a novel core–shell nanocarrier with a biodegradable property was synthesized for efficient drug release and subcellular organelle delivery. Initially, silver nanoparticles (AgNPs) were grafted with terminal double bonds originating from N, N′-bisacrylamide cystamine (BAC). Then, the outer coatings consisting of chitosan (CTS) and polyvinyl alcohol (PVA) were deposited on the surface of modified AgNPs using an emulsion method. To improve the stability, disulfide-containing BAC was simultaneously reintroduced to cross-link CTS. The as-prepared nanoparticles (CAB) possessed the desired colloidal stability and exhibited a high drug loading efficiency of cationic anticancer agent doxorubicin (DOX). Furthermore, CAB was tailored to transform their size into ultrasmall nanovehicles responding to weak acidity, high glutathione (GSH) levels, and overexpressed enzymes. The process of transformation was accompanied by sufficient DOX release from CAB. Due to the triple sensitivity, CAB enabled DOX to accumulate in the nucleus, leading to a great effect against malignant cells. *In vivo* assays demonstrated CAB loading DOX held excellent biosafety and superior antitumor capacity. Incorporating all the benefits, this proposed nanoplatform may provide valuable strategies for efficient drug delivery.

## Introduction

The advances of nanotechnology provide significant promise to reconcile the difficulties of conventional drug delivery (Mitchell et al., 2021). Until now, there have been several nanomaterials used for cancer treatment, such as liposomal doxorubicin and albumin-bound paclitaxel (Penson et al., 2020; Hama et al., 2021). However, a series of defects including poor stability, premature drug leakage, and intracellular unsatisfactory trafficking limit their application (Tan et al., 2019; Chen et al., 2020; Liu et al., 2021). Therefore, considerable therapeutic nanodevices of broad perspective are continually under development, which are generally categorized as organic nanoparticles, inorganic nanoparticles, and inorganic/organic hybrid nanosystems (Huang et al., 2020). Among these designs, the AgNP-based materials have appealed increasing attention. The popularity of AgNPs is intensively related to their unique properties, especially stable nanoscale structures, ease of synthesis, and facile surface chemistry (Rai et al., 2016; Xu et al., 2020; Yang et al., 2021).

To meet the stringent requirement for cellular delivery, AgNPs are typically subjected to surface modifications with organic polymers or biological macromolecules, allowing them for obtaining hybrid nanoparticles of several types (Sharma et al., 2014; Azizi et al., 2017; Austin et al., 2011; Schubert et al., 2018; Pedziwiatr et al., 2020). In this composite structure, AgNPs as the core take charge of size, shape, and traceability, while outer coatings determine colloidal stability, drug encapsulation efficiency, and particle elasticity. Due to the high surface energy, bare AgNPs possess poor colloidal stability, which readily aggregate or agglomerate in the blood circulation, probably causing adverse cytotoxicity (Gutierrez et al., 2020). Consequently, using coating agent for resisting the aggregation of AgNPs merits thorough consideration (Badawy et al., 2010; Hotze et al., 2010). Screening all the materials, PVA, polyethylene glycol (PEG), and poly(N-isopropylacrylamide) (PNIPAM) are excellent candidates (Stolnik et al., 2012; Jiang et al., 2016; Tzounis et al., 2019), and their appropriate compatibility, flexibility, hydrophilicity, and surface charge are highly responsible for the coating usage.

The larger size of such hybrid nanodevices, ranging from 50 to 200 nm in diameter, is beneficial for high drug loading, long circulation, enhanced permeability, and retention (EPR) effect (Duan et al., 2013). However, particles with the aforementioned size preclude subcellular organelle drug delivery because of several biological barriers (Pan et al., 2018). To overcome these limitations, a favorable drug release system (DDS) should be relatively larger in its initial size to achieve the extended circulation half-life and selective accumulation in tumor, but once entered into cells, the outer layer should detach to smaller sized particles (about 30–50 nm) to facilitate effective deep penetration, which is denoted as the “peeling onions” strategy (Ruan et al., 2015; Li et al., 2016; Niu et al., 2018). Such a requirement has promoted the recent advancement of size-transformable nanodevices. While delivering drugs to subcellular organelles, these nanodevices are capable of realizing the degradation and release smaller nanoblocks responding to the internal stimuli of cancerous cells (Tang et al., 2016; Zhou et al., 2020).

Boosting the drug release efficiency is another challenging task. Limited drug release efficiency might result in low drug levels at sites of action and thus compromise the ultimate therapeutic outcome. Therefore, more efforts should be focused on sufficient drug release. In the past few years, a multitude of individual stimulus-responsive DDSs have been developed to control drug release (Qiu et al., 2017; Xiu et al., 2020). However, single-responsive mechanisms undergo incomplete drug release and poor drug bioavailability, maximizing the administrated dosage and minimizing the treatment efficacy. Fortunately, multi-responsive DDSs are promising refinements that can promote sufficient and rapid intracellular drug release, reduce resistance in cancer cells, and garner robust therapeutic efficacy (Cheng et al., 2015; Karimi et al., 2016). It has been demonstrated that the most spectacular attributes of the intracellular milieu are hypoxia, acidic pH, high GSH levels, and overexpressed tumor-associated enzymes, which have been used as important responsive stimuli for on-demand drug release (Mi et al., 2020). Dual or triple combination of these stimuli into one nanoplatform is expected to achieve better controlled drug delivery.

CTS is naturally a kind of polysaccharide consisting of randomly distributed repeating units of both N-acetyl-D-glucosamine and N-D-glucosamine, linked through *β*-(1-4)-glycosidic bonds (Ravi Kumar et al., 2000). These structural characteristics grant CTS degradability, pH sensitivity, and enzyme sensitivity. Due to these extra properties, CTS is an extraordinary alternative for intelligent and multistage drug release (Ravi Kumar et al., 2000; Zhou et al., 2020).

**FIGURE 1:**
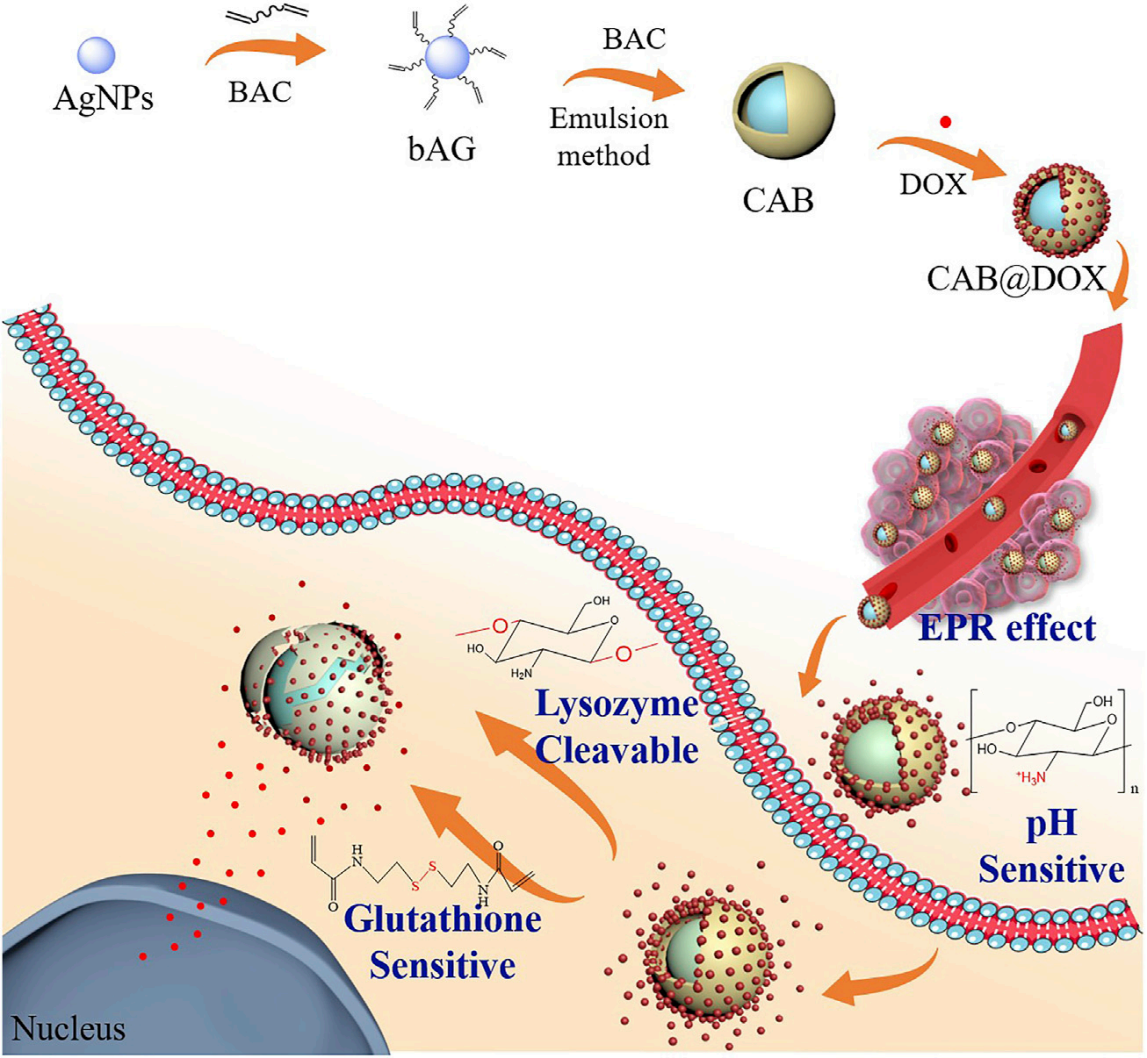
Synthesis scheme of CTS/Ag-BAC@DOX (CAB@DOX) based on chitosan and PVA coating on the AgNP surface by the emulsion method and schematic illustration of its enhanced anticancer mechanisms involving transformable size and multi-responsive drug release.

In this work, novel nanocarriers based on CTS layers surrounding AgNPs were developed (Figure 1). For immobilizing CTS, a double-cross-linking approach was used with functionalized AgNPs (forming a core–shell structure) and disulfide-containing BAC (offering redox sensitivity). PVA assembling with CTS layers during synthesis, referring to the double emulsion route, provided the engineered nanoparticles improved colloidal stability. Moreover, these nanocarriers were employed to load DOX (CAB@DOX) for cancer therapy. In the current study, CAB@DOX could respond to acidic pH, GSH, and lysozyme, causing a satisfactory drug release. In parallel, the triple responsive character also allowed CAB@DOX to shrink their size, enabling DOX preferential accumulation in the nucleus. Coupling the aforementioned properties, CAB@DOX harvested superior anticancer performance compared with free DOX.

## Materials and Methods

### Materials

Chitosan (CTS) and silver nitrate (AgNO_3_) were purchased from Sinopharm Chemical Reagent Co., Ltd. (Shanghai, China). Trisodium citrate (TSC), polyvinyl alcohol (PVA), bis(2-ethylhexyl) sulfosuccinate sodium salt (AOT), and sodium borohydride (NaBH_4_) were purchased from Aladdin Chemical Reagent Co., Ltd., (Shanghai, China). N, N′-bisacrylamide cystamine (BAC) was purchased from Alfa Aesar Chemical Co., Ltd. (Shanghai, China). Doxorubicin (DOX) was purchased from Dalian Meilun Biotechnology Co., Ltd. (Dalian, China). CAL27 cells (human oral squamous cell carcinoma cell line, OSCC) were derived from the First Affiliated Hospital of Nanchang University (Nanchang, China). Dulbecco-modified eagle medium (DMEM) and fetal bovine serum (FBS) were obtained from GIBCO Co., Ltd. (California, United States). Unless otherwise stated, the other chemical reagents were purchased from the Chinese Pharmaceutical Chemical Reagent Co., Ltd. (Shanghai, China), and the biological reagents were bought from Sigma-Aldrich (MO, United States). The abbreviations used in this article are summarized as follows: Ag-BAC (bAG), CTS/Ag-BAC (CAB), and CTS/Ag-BAC@DOX (CAB@DOX).

### Preparation of Nanoparticles

AgNPs were prepared according to the approach reported in the literature (Wang et al., 2020). In brief, TSC (29.41 mg) was mixed with NaBH_4_ (1 mg) in 40 ml of ultrapure water, and the reaction solution was stirred for 30 min at 60°C. Then, after the temperature was raised to 90°C, silver nitrate (10 mg) was added dropwise into the mixture at a pH of 10.5 and stirred for another 20 min, followed by cooling at room temperature. The spherical AgNPs were thus obtained by centrifugation at 12,000 rpm for 20 min and washed with distilled water. Next, the AgNPs and BAC (n (Ag): n (BAC) = 1:2) were mixed in the presence of sodium dodecyl sulfate (SDS) and stirred for 2 h to obtain bAG. After dialysis for 3 days using a dialysis bag (MW: 8000-14000), the obtained nanoparticle solution was preserved at 4°C.

CAB was synthesized using the emulsion method. Specifically, CTS was dissolved in acetic acid solutions (1% v/v) under continuous stirring to prepare a clear solution of chitosan (1 mg/ml). The solution is then mixed with an equal volume of bAG solution, followed by dropwise addition into the 8 ml of dioctyl sulfosuccinate sodium salt (AOT) solution in dichloromethane (2.5 wt%) and kept on stirring for 10 min. Subsequently, the mixture was added dropwise into PVA (30 ml, 2 wt%) with vigorous stirring for the second emulsification. To further cross-link CTS, 1 ml of BAC (5 mg/ml) was added into the mixture and stirred overnight. Last, the CAB solution was obtained by centrifugation at 10,000 rpm and washed 3 times, followed by preservation at 4°C. Additionally, 3 ml of the obtained nanoparticle solutions were lyophilized, respectively, to settle the amounts of nanoparticles for further experiments.

### Characterization

The morphology of different samples was detected using a transmission electron microscopy (TEM, JEM-210003040700, Japan). The hydrodynamic diameter and surface charge of different samples were tested using a particle analyzer (Nano ZS 90, Malvern). A UV−Vis spectrophotometer (V-670, Japan) was used to collect the absorbance spectra of different samples. The chemical structure of analyzed samples was studied using a Fourier Transform Infrared (FTIR) spectrometer (Bruker, Germany).

### Loading and Release of Doxorubicin

One milliliter of DOX aqueous solution at a concentration of 2 mg/ml was added to 5 ml of CAB nanoparticles solution and allowed to stir for 24 h. Then, the mixture was dialyzed against PBS with a dialysis bag (MW: 8000-14000), and the dialysate was collected 9 times. The drug amount in the dialysate was calculated based on the absorbance values. The encapsulation efficiency (EE %) of DOX was calculated according to the following formula:

$$\text{E}\text{E}\%=\frac{w_{T}-w_{D}}{w_{T}}\times 100\%$$

where *W*_*T*_ is the total content of the used DOX during the preparation and *W*_*D*_ is the amount remaining in the dialysate.

To test the breakability of these nanocarriers, CAB@DOX was suspended in a PBS solution (pH 5) of 5 mM of reduced GSH and lysozyme with a concentration of 5 μg/ml for 2 and 24 h. Next, the solution was initially sonicated for 10 minutes and then allowed for TEM analysis.

To evaluate the drug release, 1 ml of CAB loading DOX inside a dialysis bag was placed into 30 ml of PBS solutions with different pH (5.0, 6.5, and 7.4), respectively. At defined time points, 3-ml aliquots were withdrawn from the outside solution to measure its absorbance and replaced with 0.5 ml of fresh PBS solution to keep the overall volume of the solution unchanged. The percentage of DOX released from CAB@DOX (Cr %) was evaluated according to the following formula:

$${\text{C}}_{\text{r}}=\frac{w_{\text{d}}}{w_{T}}\times 100\%$$

where *W*_*d*_ is the cumulative amount of DOX released at defined time points and *W*_T_ is denoted as total DOX amount contained in CAB. For assessing the release kinetics of CAB@DOX in the absence or presence of lysozyme (5 μg/ml) and GSH (5 mM, 10 mM), the same approach was performed.

### *In Vitro* Cytotoxicity and Cellular Uptake

CAL27 cells and MTT assays were employed to evaluate the biocompatibility and toxicity of the drug. Briefly, the cells were seeded into a 96-well plate at a cell density of 5×10^5^ per well for 24 h incubation. Then, culture medium in each well was removed and replaced with 100 μL of fresh DMEM containing CAB, DOX, and CAB@DOX. The concentration of DOX (2.0 μM) in the DOX group and CAB@DOX group were adjusted to equalization before being fed into the well. After incubation for another 24 h, the cells were washed 3 times, and 10 μL of MTT (5 mg/ml) was added to each well for further 4 h incubation. Finally, the cells without any medium were incubated with 150 μL of DMSO for 10 min. The corresponding absorbance per well was detected using a microplate reader at 490 nm. The relative viability (CV %) was calculated by the following formula:

$$CV\%=\frac{OD_{eg}}{OD_{cg}}\times 100\%,$$

where OD_eg_ is the OD value for the experimental group and OD_cg_ is the OD value for the untreated control group.

To evaluate the cell survival and death, CAL27 cells were seeded into the 20-mm CLSM cell culture dish. After the cell confluence reached approximately 70%, the initial medium was replaced by fresh PBS solution containing DOX and CAB@DOX for 24-h incubation, respectively, in which the concentration of DOX was maintained at 2 μM. Then, these treated cells were stained with AO/EB for 15 min and imaged under a confocal microscope (CLSM, Nikon C2, Japan).

To demonstrate the superiority of CAB in aiding DOX to enter cells, the efficiency of cellular uptake in the free group and CAB@DOX group was evaluated. CAL27 cells were seeded into the 20-mm CLSM cell culture dish at a density of 1×10^6^ cells. After incubation for 24 h, DOX and CAB@DOX solutions with an equivalent DOX concentration (2 μM) were fed into the dish for further incubation. Later, the solutions were removed at the time points of 4 and 24 h, followed by washing 3 times, and fixing with 4% paraformaldehyde for 30 min. Next, the cells were washed 3 times again and fixed with DAPI for 5 min, followed by washing 3 times. Finally, the samples were subjected to observations with CLSM.

### *In Vivo* Antitumor Studies

Nude mice were brought from Charles River Experimental Animal Co., Ltd. (Beijing, China). All animals were used and handled in accordance with the Guidelines for the Care and Use of Laboratory Animals of Nanchang University. The xenograft OSCC model was established by injecting 100 μL of PBS containing 1×10^7^ CAL27 cells subcutaneously in the right side of the oxter of the nude mice. After the tumor volume reached 150 mm^3^, the experimental nude mice were randomly allocated into three groups. Each group was injected intraperitoneally with 1 ml of three kinds of solution (PBS, DOX, CAB@DOX). The dosage of DOX was 4 mg kg^−1^ for each mouse. The tumor volume and body weight were determined daily. After 21 days, the heart, liver, spleen, lung, and kidney together with tumor were harvested, followed by fixing with 4% paraformaldehyde solution and sectioning. Next, the tissue slides were stained with hematoxylin and eosin (H&E) in accordance with the standard procedure (Liu et al., 2018). Furthermore, the tissue slides were analyzed using terminal deoxynucleotidyl transferase-mediated dUTP-biotin nick-end labeling (TUNEL) assays (Sharma et al., 2021) to evaluate the antitumor efficacy. Furthermore, blood samples of nude mice with different treatment were collected for biochemical analyses on the 21st day.

### Statistical Analysis

Data were presented as mean ± standard deviation. The differences among the independent groups were evaluated by using a two-tailed unpaired Student’s t-test, and the differences between multiple groups were evaluated by the one-way analysis of variance (ANOVA). *p < 0.05, **p < 0.01, and ***p < 0.001 were used to express the level of significance.

## Results and Discussion

### Preparation and Characterization of Nanoparticles

In contrast to gold nanoparticles, AgNP perhaps is a more valuable nanomaterial on account of lower cost and higher reactivity, which widens the accessibility of surface functionalization (Desireddy et al., 2013). Recently, great efforts were spent to utilize the AgNPs as vehicles for cancer treatment (Zeng et al., 2018; Gomes et al., 2021). To optimize colloidal stability and drug loading, AgNPs are commonly subjected to surface modification with natural macromolecules which embrace outstanding biocompatibility (Akter et al., 2018; Gutierrez et al., 2020). As a representative material of natural polysaccharides, CTS has been approved by the U.S. Food and Drug Administration (FDA) employed in a variety of foods. Of note, CTS possessing the properties of intelligence and biodegradability makes them available for drug delivery, especially in the form of nanogel (Divya et al., 2017; Jafari et al., 2021).

**FIGURE 2:**
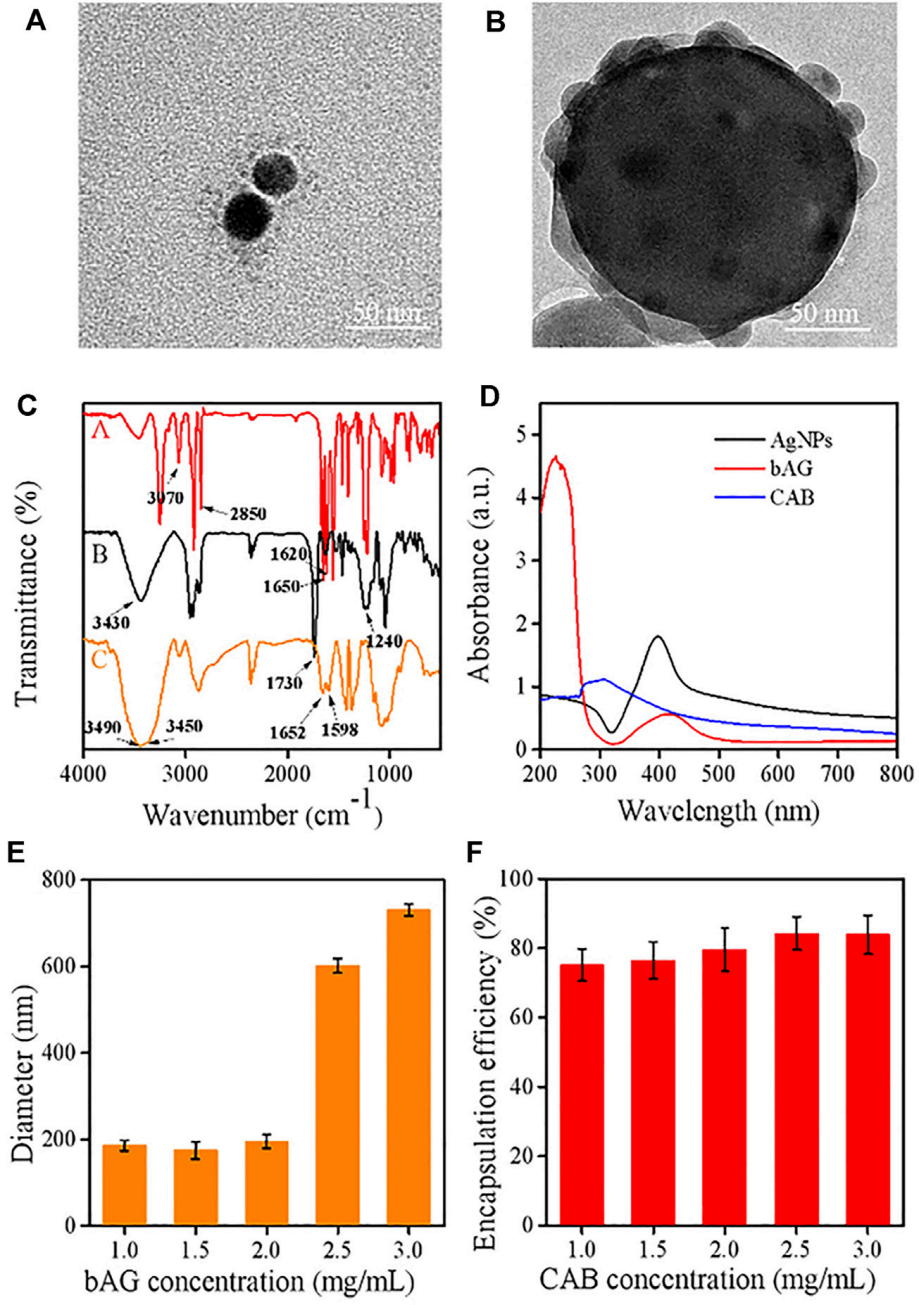
TEM images of **(A)** bAG and **(B)** CAB; **(C)** FT-IR spectra of various samples [bAG **(A)**, CAB **(B)**, and CTS **(C)**]; **(D)** UV–visible absorption spectra of AgNPs, bAG, and CAB; **(E)** effect of bAG concentration on the CAB size; **(F)** effect of CAB concentration on the DOX encapsulation efficiency.

In this work, a type of hybrid AgNPs was prepared by coating the CTS shell containing PVA. Bare AgNPs were initially synthesized which exhibited homogeneous dispersion and size distribution around 36 ± 3 nm (Supplementary Figure S1). Subsequently, with the cleavage of the disulfide linkage of BAC, the cystamine units containing terminal carbon–carbon double bonds were anchored onto the surface of AgNP through the Ag-S covalent bond (Huang et al., 2021). The TEM image showed that the size of bAG was slightly larger than AgNPs (Figure 2A), supporting the successful modification. Then, the modified AgNPs allowed for cross-linking CTS through the Michael addition reaction between -C=C and -NH_2_ of CTS. To enhance the structural stability, BAC was reintroduced to react with CTS, resulting in the formulation of double-cross-linked nanoparticles (CAB). Due to the existence of disulfide bonds, CAB were sensitive to the reductive environment. The TEM analysis of the CAB showed that AgNPs were densely encapsulated by CTS (Figure 2B). Furthermore, the preparation process followed a double emulsion method. The emulsifier, PVA, used in this process was able to self-assemble with chitosan through electrostatic interactions. Figure 2B provides visual evidence of successful introduction of PVA as contrast could be observed in the outmost layer.

The colloidal stability of metallic nanoparticles is a concerning factor, which affects their application potential in the biomedical field. In case of noble metals, AgNPs are the most likely to aggregate. While used in organisms, the aggregates might not only lead to accelerated elimination by the reticuloendothelial system but also cause severe toxicity. Therefore, the colloidal stability of CAB was investigated by using the dispersion test in Ultra-water, DMEM, and PBS, respectively. During the 7-day observation, there was no obvious precipitation for the three groups (Supplementary Figure S2), demonstrating the coating agent could well alter the surface attributes of AgNPs and help remove a key obstacle of AgNPs for clinical translation.

FTIR measurements were used to manifest the successful modification of AgNPs. In the spectrum of bAG, two characteristic peaks appeared at 1,650 cm^−1^ and 1,620 cm^−1^, which were attributed to -C=C- stretch vibration and -C (=O)-NH_2_ stretch vibration, respectively. These results suggested successful modification of AgNPs with cystamine unit (Wang et al., 2020; Jafari et al., 2021). Moreover, the FTIR spectrum of CTS exhibited peaks located at 3,490 cm^−1^ and 3,450 cm^−1^, while a new peak presented at 3,430 cm^−1^ in CAB, indicating primary amines transformed into secondary amines. Meanwhile, the peak at 1,420 cm^−1^ in the spectrum of CAB was assigned to the formation of -C-N- bonds, which implied the occurrence of Michael’s addition reaction (Figure 2C). In UV/Vis absorption spectrum, the maximum absorption wavelength in AgNPs and bAG were, respectively, at 400 and 426 nm. The red shift of peak was related to the increase in diameter and thus confirmed successful conjunction of the cystamine unit (Figure 2D).

The circulation time determines the ultimate fates of nanoscale drugs, in which particle size and surface charge play a vital role (Patra et al., 2018). In the research setting, a suitable diameter for extending the circulation and targeting tumor accumulation should be less than 200 nm. Therefore, the effects of CTS and bAG at different concentrations on diameters were tested by DLS. It could be observed that the higher was the concentration of CTS, the smaller was the particle size (Supplementary Figure S3A). This result might be ascribed to the higher concentration of CTS that made the nanoparticles more compact. As shown in Figure 2E, the size of CAB significantly increased in pace with the rise of bAG concentration restricted to 1.0 ∼ 3.0 mg/ml. Specifically, adjusting the bAG concentration to 1.0 mg/ml resulted in a diameter of 175 ± 3 nm. Furthermore, the surface charge of nanoparticles affects the adsorption of opsonin and subsequent elimination by macrophages. In pharmacokinetics, the negatively charged nanoparticles reduce the likelihood of phagocytic uptake, facilitating circulation time elongation. Although coated with cationic CTS, CAB showed a negative surface charge. The relationship between CAB potential and bAG concentration is shown in Supplementary Figure S3B. At a high bAG concentration, the value of potential was low, which might be attributed to the additional introduction of negatively charged bAG. More importantly, the shell layer accomplished a high loading of cationic DOX at up to 80% encapsulation efficiency, which is favorable for the therapeutic effect. As positively charged CTS possesses poor cationic drug absorption, this nanoplatform might broaden the range of loaded drugs for CTS (Figure 2F).

### Drug Release and Degradation Assays *In Vitro*

**FIGURE 3:**
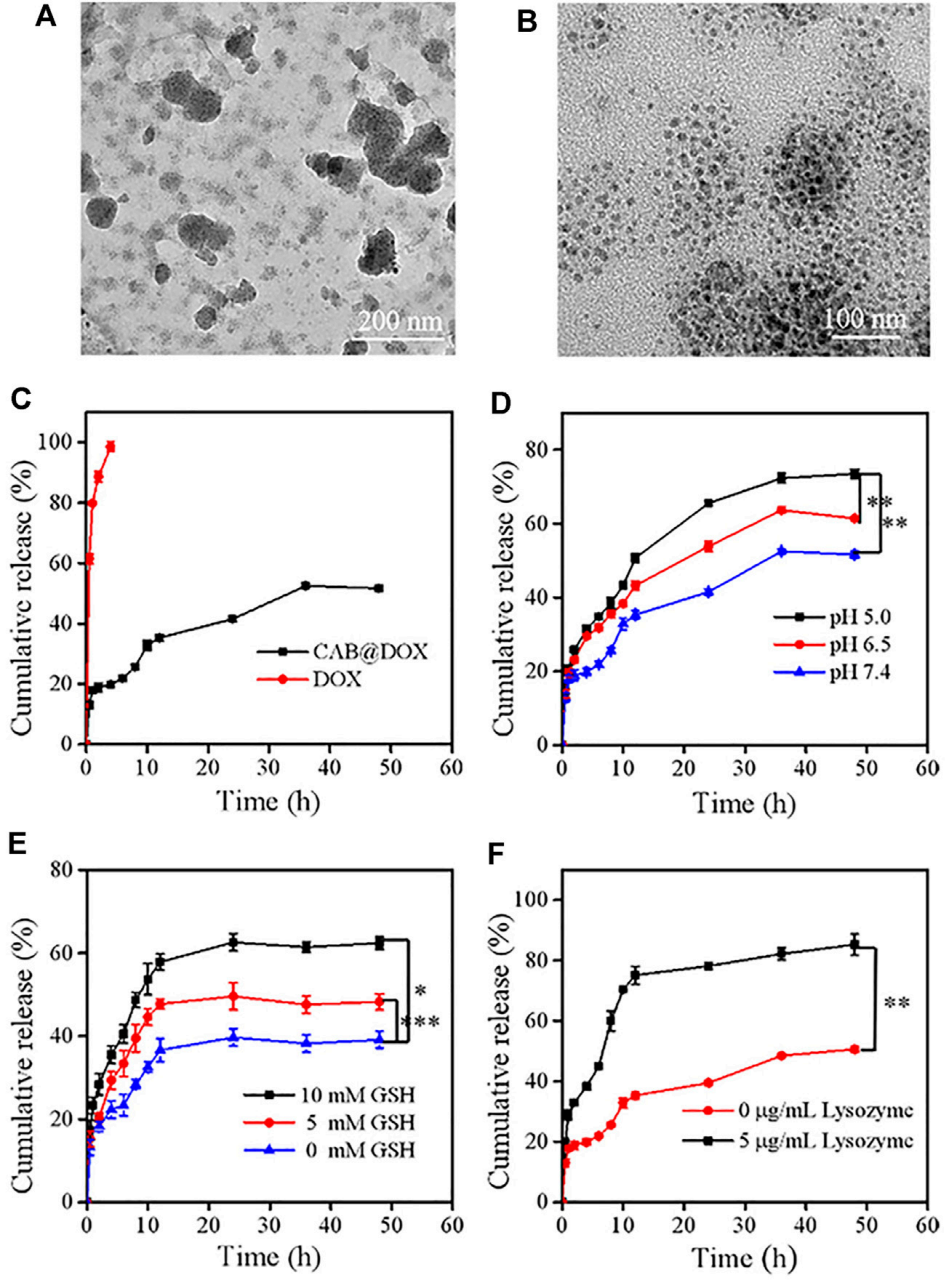
Degradation and release behaviors of CAB@DOX in a stimulation environment *in vitro*. The degradation TEM of CAB@DOX in pH 5, 5 mM GSH, and 5 μg/ml lysozyme of PBS solution for **(A)** 2 h and **(B)** 24 h. **(C)** Release profiles of DOX, CAB@DOX in PBS buffer (pH 7.4) at 37°C; **(D)** profiles of DOX release from CAB at pH values of 5.0, 6.5, and 7.4; **(E)** release profiles of DOX in the presence of different concentrations of GSH (0 mM, 5.0, and 10.0 mM); **(F)** release profiles of DOX in the absence or presence of 5 μg/ml of lysozyme.

The initial size of AgNPs was 36 nm and increased to around 175 nm after being decorated with CTS. To evaluate the shrinkable capacity, CAB@DOX was incubated in PBS (pH 5.0) with GSH (5 mM) along with lysozyme (5 μg/ml) analog to cancerous cells. After being treated for 2 h, the particles showed decreased size and inhomogeneous distribution (Figure 3A), suggesting that nanoparticles could disassemble. This result was presumably due to the cleavage of intramolecular *β*-(1-4)-glycosidic bonds and disulfide bonds in CTS and BAC, while encountering such a microenvironment. At a time interval of 24 h, the variation of CAB@DOX was extremely marked, and many smaller particles were obtained ranging at 10–20 nm (Figure 3B). It has been reported that smaller CTS nanoparticles (<39 nm) had a greater chance of localizing in the nucleus (Tammam et al., 2015). CAB@DOX with such size-variable characteristics was consequently beneficial for targeting nucleic delivery of DOX.

The release behavior of DOX, which significantly affects its therapeutic bioactivity, was investigated. Generally, ideal nanodevices should recognize especially the tumor microenvironment, speeding up drug release selectively. Also, they should avoid premature drug leakage under physiological conditions which may lead to off-target effects. Detected by the dialysis approach, free DOX molecules could permeate through the dialysis membrane easily and CAB could heavily suppress the release of DOX as a diffusion barrier (Figure 3C). Considering the breakability of CAB@DOX, the unique conditions of tumor tissue could readily trigger the release of DOX. Compared with neutral conditions (pH 7.4), DOX release tended to be much faster during the first 12 h responding to a weakly acidic environment, representing neoplastic interstitial microenvironment (pH 6.5) and endo/lysosomes (pH 5.0) (Figure 3D) (Bazban et al., 2017). This difference was profoundly related to ionization of amino groups and the swelling up of CTS (Ahmed et al., 2016). Furthermore, the concentration of GSH in tumor cells (2–10 mM) is 10 times more than that of normal tissue (2–20 μM) (Tu et al., 2017), causing a high redox state. Clearly, upon facing the high level of GSH, the drug release was boosted, reaching about 60% for 15 h (Figure 3E). The introduction of disulfide bonds might contribute to accelerated drug release in a reductive environment. Additionally, the enzymes (e.g., lysozyme, phospholipase, and glycosidase) are a fascinating element to design smart DDSs as they are often overexpressed in tumor tissues for cell growth, invasion, and metastasis (Li et al., 2017; Zhou et al., 2020). The cumulative release ratio of DOX in the presence of lysozyme was 80% after 12 h which was 2-fold as high as that in the absence of lysozyme (Figure 3F). Nearly all the loaded drug released might be a consequence of the degradation of CAB. By lysozyme, CAB could vary to one hundred smaller sized nanocarriers through the cleavage of *β*-(1-4)-glycosidic bonds, accompanied with massive drug release. The aforementioned experimental results fully demonstrated CAB@DOX embraced good stability under physiological conditions, lowering unwanted side effects. Furthermore, CAB@DOX experienced sufficient drug release responding to stimulated malignant cell conditions featuring weak acidity, high glutathione (GSH) levels, and overexpressed lysozyme. It was expected the triple responsive nature would give rise to a high intracellular drug level.

### *In Vitro* Cytotoxicity and Growth Inhibition in CAL27 Cells

**FIGURE 4:**
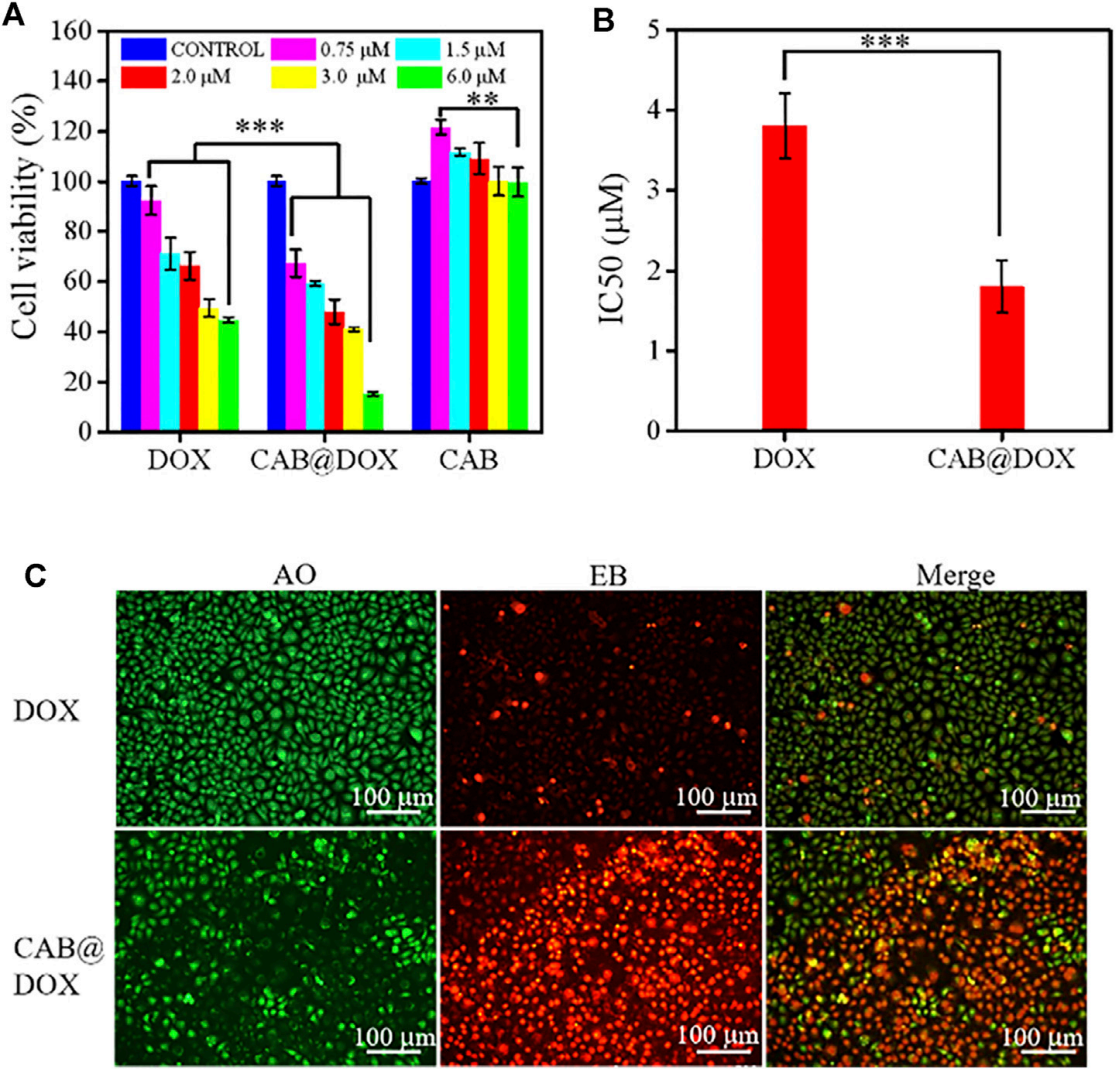
*In vitro* biocompatibility and growth inhibition in CAL27 cells. **(A)** Cell viability of CAL27 cells cultured with different concentration of DOX, CAB@DOX, and CAB for 24 h; **(B)** IC50 value of DOX and CAB@DOX; **(C)** AO/EB staining fluorescence images of CAL27 cells treated with DOX and CAB@DOX for 24 h.

The capability of CAB@DOX to induce cellular apoptosis was evaluated. CAL27 cells were incubated with free DOX and CAB@DOX at different DOX concentrations. In the assays, the concentration of CAB@DOX was expressed based on the DOX content. As shown in both groups, an apparent trend of reduced cell viability was observed at an increasing DOX concentration from 0.75 to 6 μM (Figure 4A). Furthermore, in contrast, the proportion of non-viable cells treated with CAB@DOX was larger in all studied concentrations. These results were likely related to high drug loading efficiency and on-demand drug release of CAB@DOX, which caused a corresponding increase in the availability of DOX to targets of action. The grand challenge in AgNP design for drug delivery lies in toxicity (Caballero et al., 2013). For clinical adoption, it is critical to develop many safety AgNP-based vectors. Since CTS and PVA are fascinating biocompatible materials and could render AgNPs a hydrophilic surface to resist aggregation, this nanohybrid might have reduced toxicity. As anticipated, CAL27 cells showed no signs of loss in cell viability after incubation with empty CAB even at a high concentration (Figure 4A), suggesting that CAB alone was biocompatible and itself did not contribute to the anticancer effect of this transporter–drug conjugate. Furthermore, IC50 of CAB@DOX in the CAL27 cell line at 24 h was 1.9 μM, which was 2-fold less than that of free DOX (∼3.8 μM) (Figure 4B). Next, more intuitive cytotoxicity assays were conducted by using an AO/EB double staining experiment. Remarkably, most cancerous cells were killed (orange fluorescence) in the CAB@DOX group, while large amounts of cells remained alive (green fluorescence) treated with free DOX (Figure 4C). The IC50 value and AO/EB assays further confirmed the great potential of CAB aiding in DOX to implement potent anticancer activity.

### *In Vitro* Cellular Uptake

**FIGURE 5:**
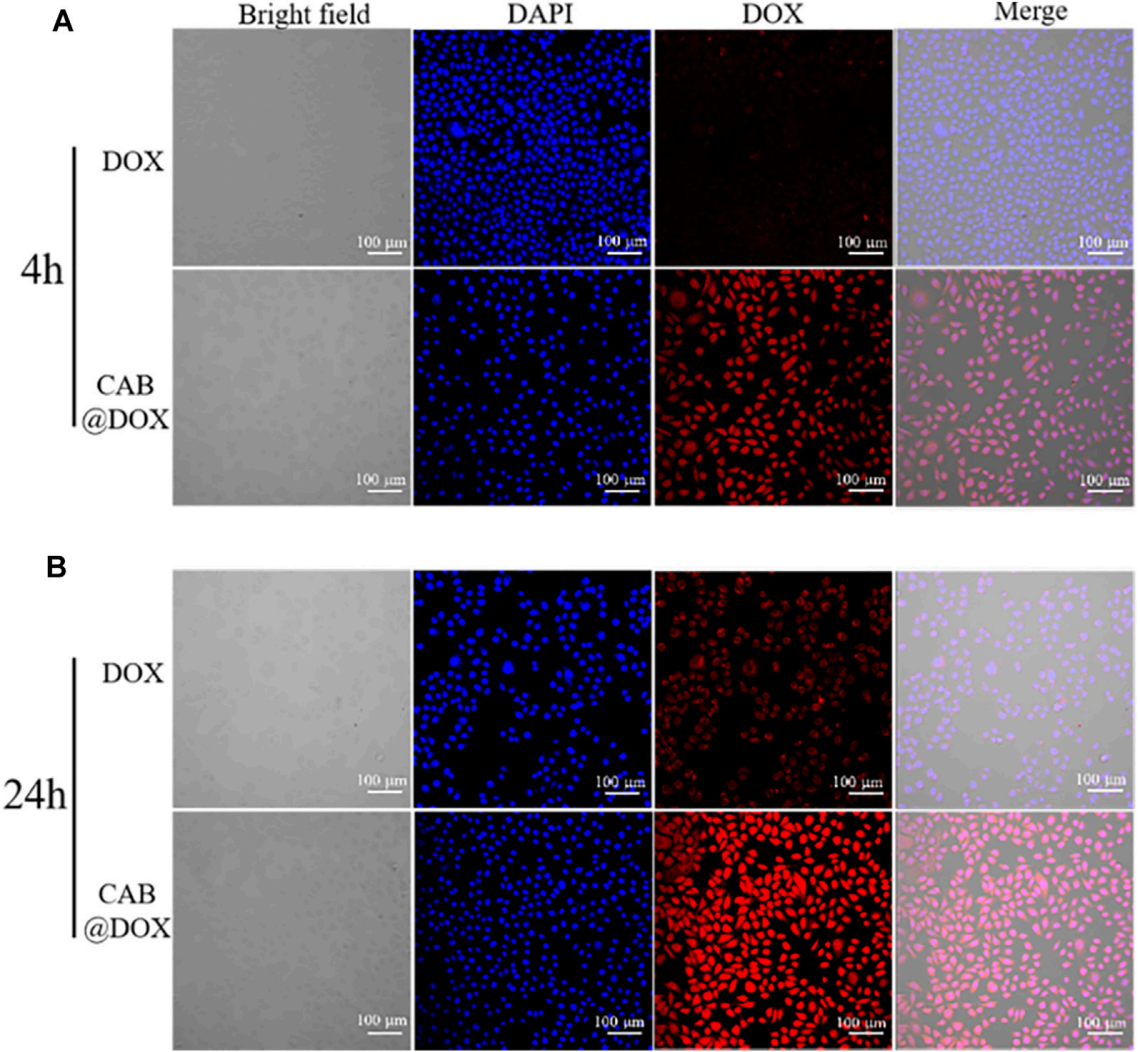
Confocal images of CAL27 cells after incubation with free DOX and CAB@DOX for **(A)** 4 h and **(B)** 24 h.

The potential mechanism of antitumor activity was further elucidated by CLSM. As the nucleus is the main interaction site of DOX, the capability of reaching the nucleus was highlighted. In the first 4-h incubation, DOX alone entered cells slowly as red fluorescence signals were drastically weak. However, strong red fluorescence was observed and overlapped extensively with nuclei in the CAB@DOX group (Figure 5A). The rapid cellular uptake of CAB@DOX putatively resulted from CAB@DOX passing through the cell membrane in a manner of endocytosis, which was more efficient than passive diffusion of free DOX (Wang et al., 2012). When increasing the incubation time to 24 h, more DOX entered the cells and enriched the nuclei in the CAB@DOX group, indicating the cellular uptake was in a time-dependent pattern. In sharp contrast, few DOXs were seen in the cytoplasm. Although intracellular DOX in the free DOX group increased, it is mainly located at the peri-nucleus (Figure 5B). DOX are internalized into nuclei as soon as they enter the cells in the CAB@DOX group, which might be due to the transformable capability of CAB. In nucleus-targeting nanotherapeutics, creating a transformable vector is the most prevalent resolution as the needs for long circulation time and subcellular drug trafficking are contrary design elements. Specifically, the large particle size avoids excretion by the kidney, while the small size allows for intercellular deep drug delivery. It has been demonstrated nanoparticles of 40 nm diameter enable faster diffusion into the nucleus (Pan et al., 2018). In light of degradation assay, in the cytoplasm, CAB@DOX is capable of responding to the pathological environment, emits smaller sized carriers (less than 40 nm), and consequently concentrates DOX in the nucleus. Therefore, CAB@DOX can exhibit a better inhibitory effect on tumor cells than most DOX-loaded carriers (e.g., mesoporous silica (MSN), carbon nanotubes (CNTs), and hydroxyapatite (HAp)) as the latter could not transport DOX to the site of action (Dong et al., 2017; Llinas et al., 2018; Ghosh et al., 2020).

### *In Vivo* Antitumor Activity

**FIGURE 6:**
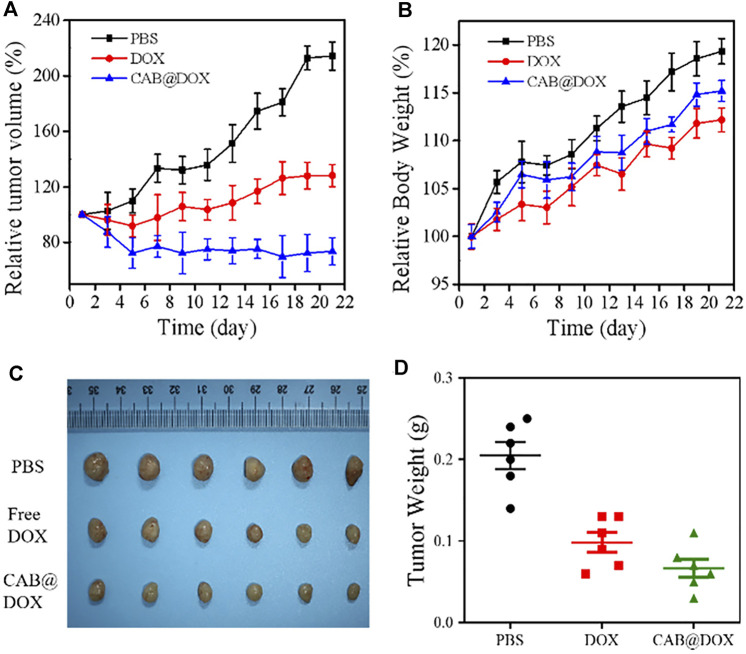
*In vivo* antitumor activity of CAB@DOX in OSCC-bearing mice. **(A)** Relative tumor volume in tumor-bearing mice; **(B)** Relative body weight of the tumor-bearing mice; **(C)** representative images of resected tumors after 21 days of various treatment; **(D)** tumor weight of nude mice.

The antitumor efficacy of CAB@DOX *in vivo* was evaluated during the 21-day treatment process. These mice were allocated into three groups randomly (*n* = 6): PBS, DOX, and CAB@DOX. As expected, the tumor volume of the PBS-treated mice increased rapidly, whereas the tumor growth of the nude mice in the free DOX group and CAB@DOX group was markedly inhibited (Figure 6A). In particular, the treatment efficacy in the CAB@DOX group was much more apparent than that of the DOX group, and the difference between the two groups could reach 3 times. On day 21, all the mice were killed for tumor collection. It was found that CAB@DOX displayed much higher inhibition efficiency against tumor growth than free DOX (Figures 6C,D). Based on the results, it could be demonstrated the great potential of CAB@DOX against OSCC which resulted from enhanced cellular uptake, stimulus-responsive drug release, and higher drug accumulation in nuclei. Furthermore, the fluctuation in body weight is a direct indicator of drug toxicity. Compared with the control, no meaningful difference of body weight change was observed in the CAB@DOX-treated mice over the course of treatment, which might be related to the fact that CAB@DOX effectively reduced side effects caused by DOX (Figure 6B).

**FIGURE 7:**
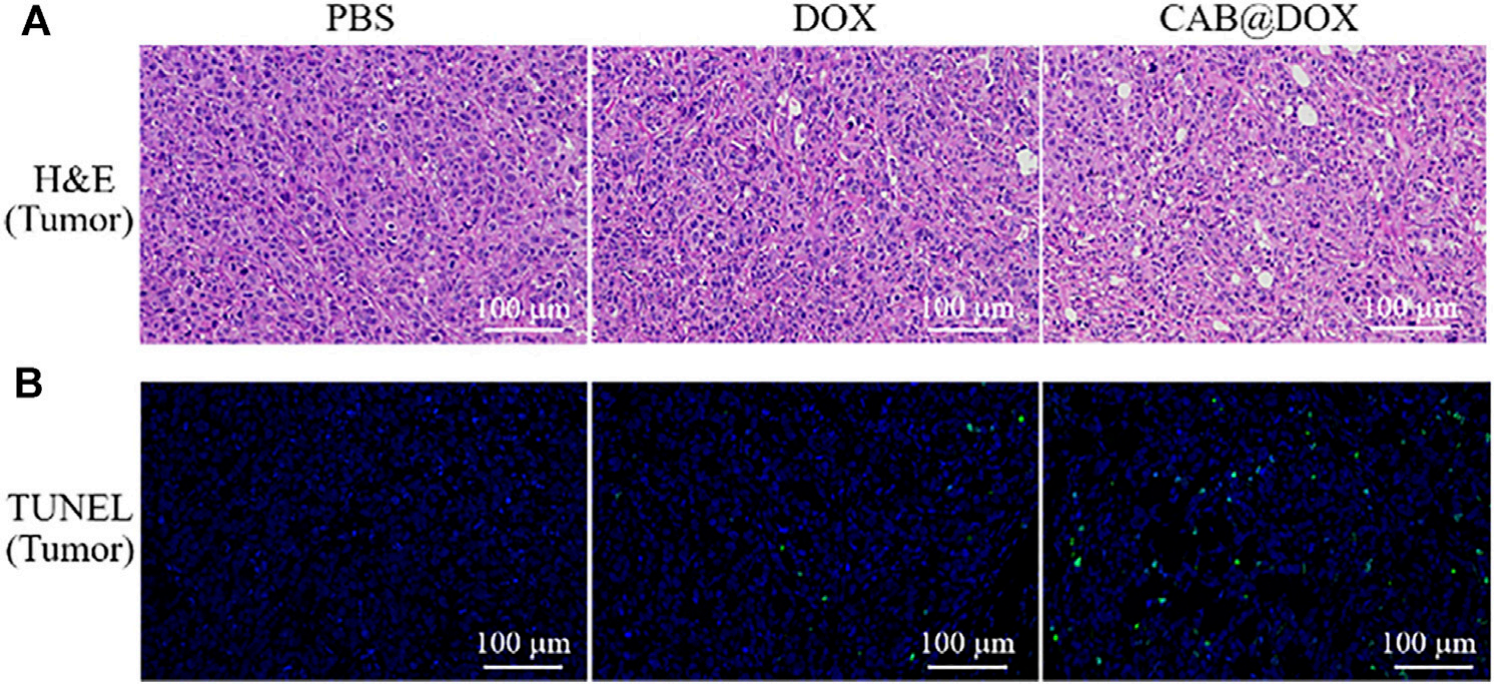
**(A)** H&E staining of tumors; **(B)** apoptotic cell detection by TUNEL immunofluorescence staining.

To further confirm the therapeutic outcome *in vivo*, H&E staining and TUNEL assays were performed. As displayed in Figure 7A, severe cancerous cell damage (pyknosis, fragmentation, and lysis) was observed by H&E staining in the CAB@DOX group, which was consistent with the results of the relative tumor volume study. Similarly, TUNEL assays were carried out on the resected tumors, which is a general method to assess apoptotic cells in tumors. Figure 7B shows that CAB@DOX induced significant apoptosis in the tumors, while DOX-treated tumors showed an obvious low rate of apoptosis. Therefore, it could be confirmed that DOX loaded in the CAB was more likely to eliminate cancerous cells than systemic direct administration.

**FIGURE 8:**
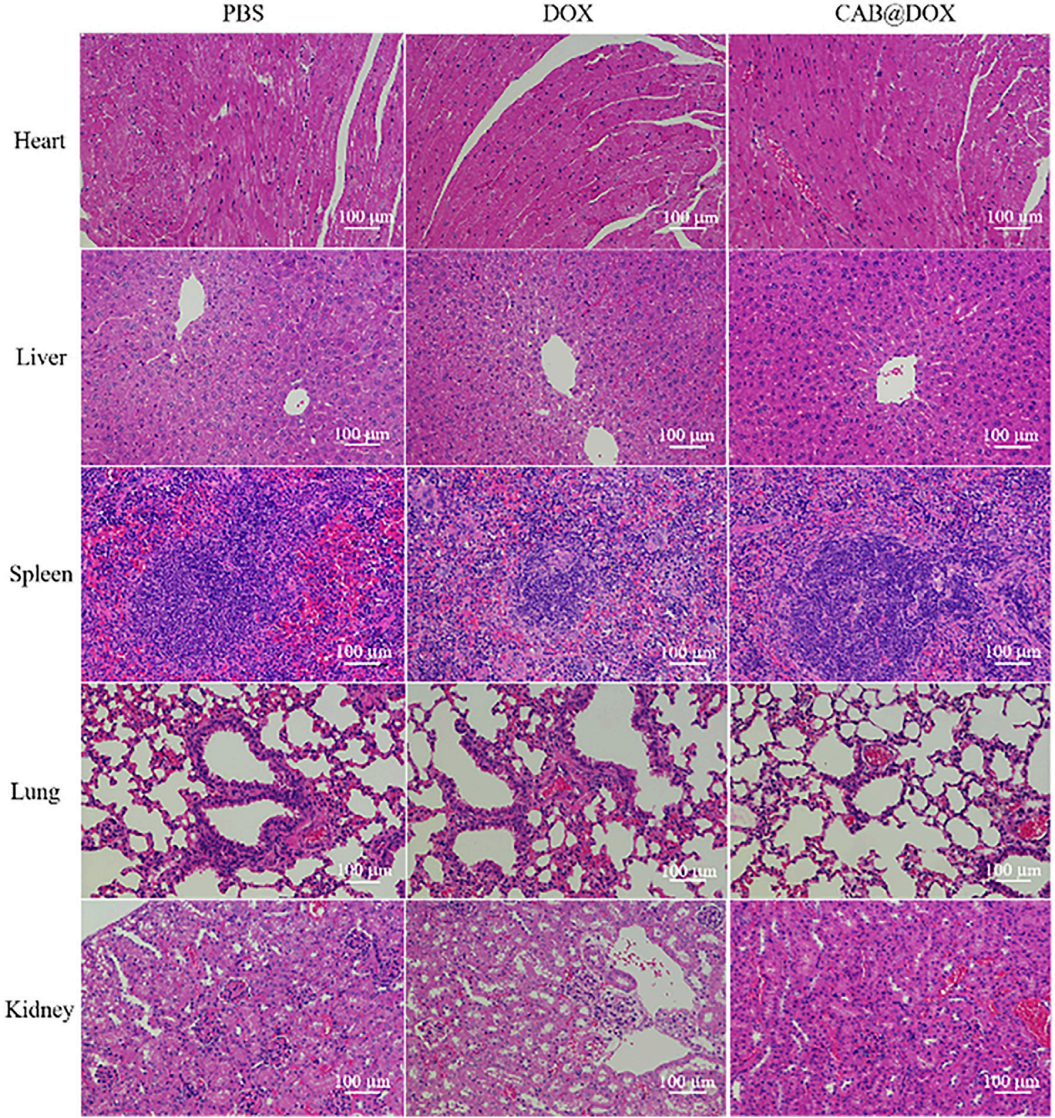
Histological analysis of the vital organs (heart, liver, spleen, lung, and kidney) of mice treated with PBS, DOX, and CAB@DOX, respectively.

**FIGURE 9:**
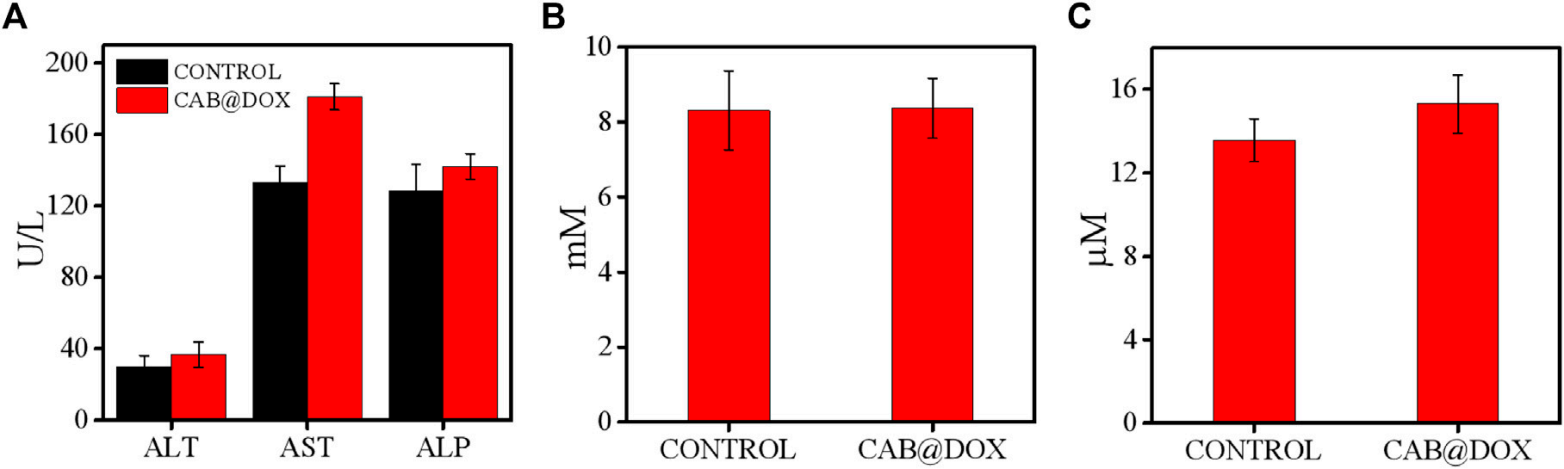
Blood biochemistry indicators including **(A)** ALT, AST, ALP, **(B)** BUN, and **(C)** CRE with or without CAB@DOX, respectively (*n* = 6).

The potential toxicity of DOX, also displayed by the change of body weight, impedes its clinical use. Under the results of assays *in vitro*, good parameters for colloidal stability, and selective drug release, CAB@DOX might imply a low biotoxicity. Herein, the biosafety of CAB@DOX was further investigated. All mice were killed at day 21 posttreatment, followed by capturing photographs (Supplementary Figure S4), and the H&E staining exhibited no off-target damage to vital organs (liver, heart, lung, kidney, and spleen) (Figure 8). Additionally, the blood of the killed mice was collected for blood biochemistry analysis. Eventually, no significant differences were found between CAB@DOX-treated groups and the control group in the blood biochemistry indicators (i.e., alanine transaminase (ALT), aspartate aminotransferase (AST), alkaline phosphatase (ALP), blood urea nitrogen (BUN), and creatinine (CRE)) (Figure 9). These outcomes indicated CAB@DOX had negligible side effects and could effectively lower the heart and kidney damage induced by DOX. Although the current work revealed the potential of CAB in improving drug safety, there was still needed more assays to systematically study long-term toxicity.

## Conclusion

In summary, we rationally developed a core–shell nanocarrier. AgNPs were used as the core, with a well-defined size of around 175 nm. The coatings consisting of CTS and PVA stabilized particles against agglomerates, offering a novel way for developing AgNP-based materials in biomedicine. Moreover, the final nanoparticles, CAB, exhibited high DOX encapsulation efficiency for 80%, which is favorable for antitumor efficacy. While encountering the environment analogous to a cancerous cell, CAB disassembled and emitted smaller sized nanoblocks (10–20 nm). Meanwhile, shrunk nanoparticles allowed for accelerated DOX release and thus help achieve desired drug bioavailability. Due to these properties, DOX loaded in CAB is easily enriched in the nucleus, leading to an obvious inhibitory effect toward CAL27 cells. Using a human oral squamous cell carcinoma-bearing mouse model, CAB@DOX exhibited an enhanced antitumor efficacy than free DOX while showing a negligible influence on normal cells and tissues. Taken together, the nanohybrid is a promising vehicle for cancer therapy which can deliver other types of drugs in the future.

## Data Availability

The original contributions presented in the study are included in the article/Supplementary Material, further inquiries can be directed to the corresponding author.
