# Supplementary material for: pH/Redox/Lysozyme-Sensitive Hybrid Nanocarriers With Transformable Size for Multistage Drug Delivery
Source: Front Bioeng Biotechnol. 2022 Apr 11;10:882308. doi: 10.3389/fbioe.2022.882308 (PMC9035699; doi:10.3389/fbioe.2022.882308)
Supplement: Supplementary file 1 [file DataSheet1.doc]

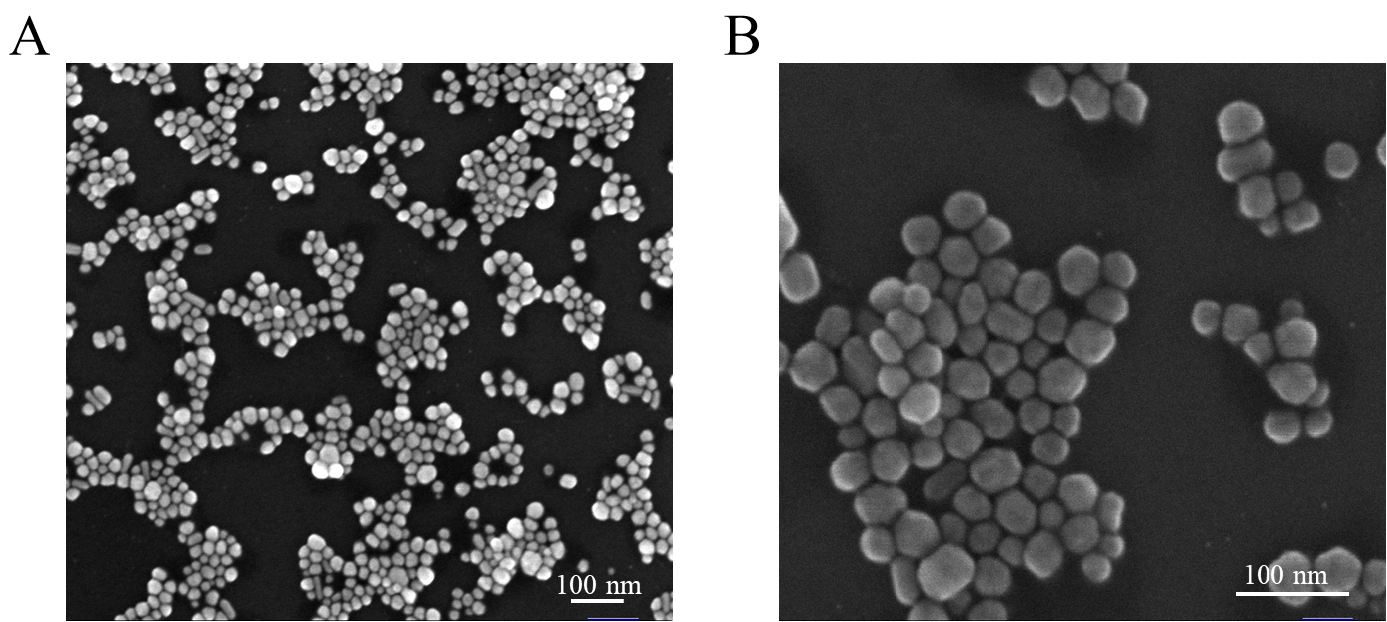


**Figure S1.** SEM images of Ag NPs.


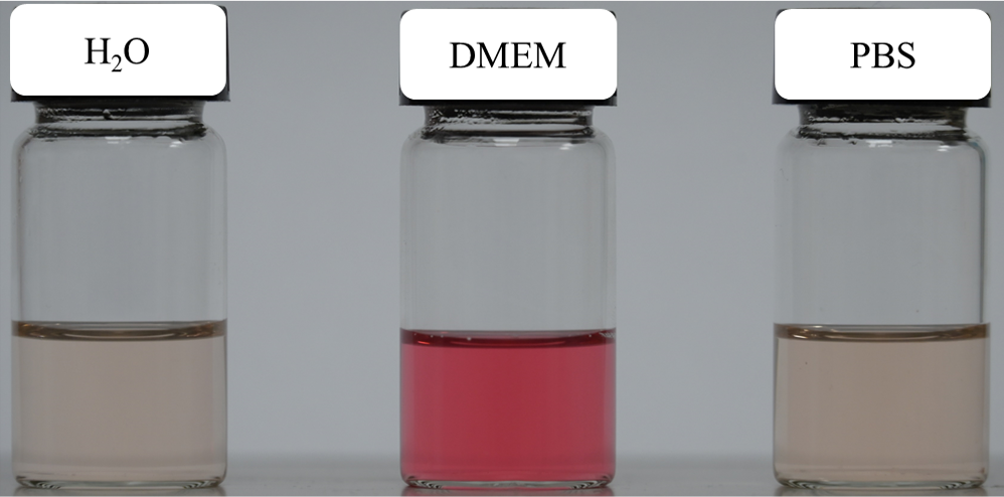


**Figure S2.** Photo of CAB preserved in Ultra-water, DMEM, PBS for 7 days.


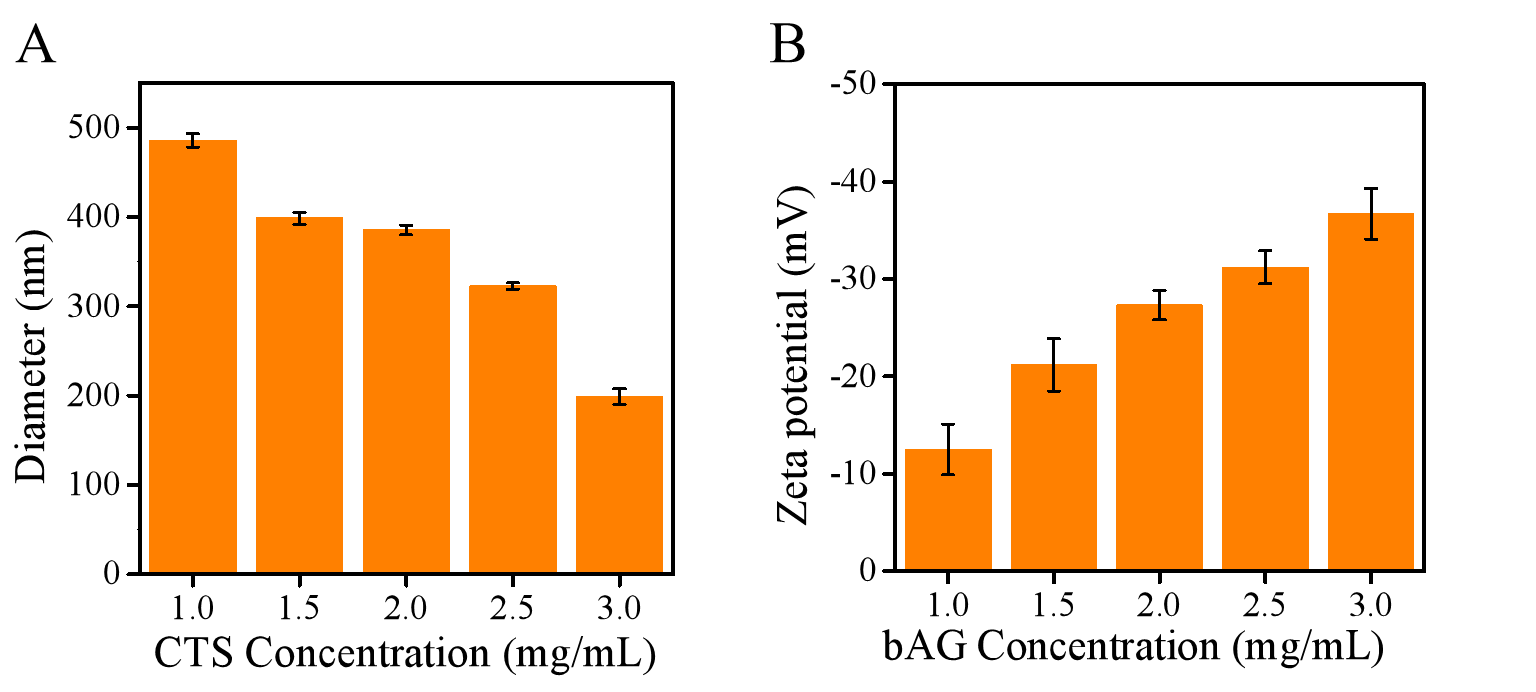


**Figure S3.** (A) Effect of CTS concentration on the CAB size; (B) Effect of bAG concentration on zeta potential of CAB.


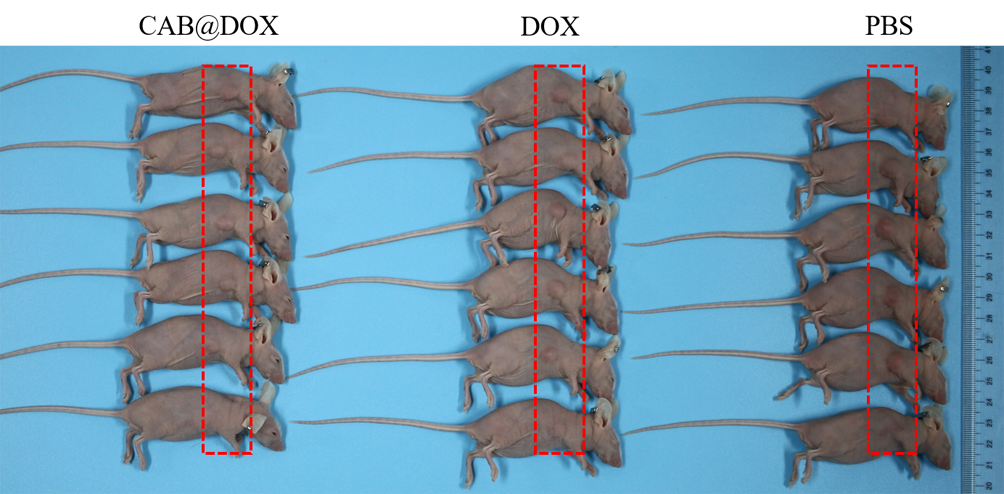


**Figure S4.** Optical photo of the tumor-bearing mice administrated with CAB@DOX, DOX, PBS at day 21 post treatment.
